# Lindsay & Blakiston’s Visiting List

**Published:** 1874-12

**Authors:** 


*Lindsay & Blackiston's Visiting List for* 1875.

The Visiting List of Lindsay & Blackiston has so long been the favorite with the profession that it needs no notice at our hands, except the mention that it has put in its appearance, in its familiar form, for 1875.

